# The Role of Regulatory CD4 T Cells in Maintaining Tolerance in a Mouse Model of Autoimmune Hepatitis

**DOI:** 10.1371/journal.pone.0143715

**Published:** 2015-11-24

**Authors:** Ira an Haack, Katja Derkow, Mathias Riehn, Marc-Nicolas Rentinck, Anja A. Kühl, Seija Lehnardt, Eckart Schott

**Affiliations:** 1 Dept. of Hepatology and Gastroenterology, Charité Universitätsmedizin Berlin, Berlin, Germany; 2 Institute of Cell Biology and Neurobiology, Center for Anatomy, Charité Universitätsmedizin Berlin, Berlin, Germany; 3 Model Systems for Infection and Immunity, Helmholtz Centre for Infection Research Braunschweig, Braunschweig, Germany; 4 Dept. of Medicine I for Gastroenterology, Infectious Diseases and Rheumatology/Research Center ImmunoSciences, Charité Universitätsmedizin Berlin, Berlin, Germany; 5 Cluster of Excellence NeuroCure, Charité Universitätsmedizin Berlin, Berlin, Germany; 6 Department of Neurology, Charité Universitätsmedizin Berlin, Berlin, Germany; University of Lisbon, PORTUGAL

## Abstract

**Background:**

The role of regulatory CD4 T cells (Treg) in immune-mediated liver disease is still under debate. It remains disputed whether Treg suppress T cell-mediated hepatitis *in vivo* and whether hepatic regulatory T cells are functional in patients with autoimmune hepatitis.

**Methods:**

We used TF-OVA mice, which express ovalbumin in hepatocytes, to investigate the impact of Treg in a model of autoimmune hepatitis. Treg isolated from inflamed livers of TF-OVA mice were tested for their functionality *in vitro*. By employing double transgenic TF-OVAxDEREG (DEpletion of REGulatory T cells) mice we analyzed whether Treg-depletion aggravates autoimmune inflammation in the liver *in vivo*.

**Results:**

CD25^+^Foxp3^+^ CD4 T cells accumulated in the liver in the course of CD8 T cell-mediated hepatitis. Treg isolated from inflamed livers were functional to suppress CD8 T-cell proliferation *in vitro*. Depletion of Treg in TF-OVAxDEREG mice dramatically amplified T cell-mediated hepatitis. Repeated administration of antigen-specific CD8 T cells led to a second wave of inflammation only after depletion of Treg.

**Conclusion:**

Our data add to the evidence for an important role of Treg in autoimmune hepatitis and show that Treg reduce the severity of T-cell mediated hepatitis *in vivo*. They constitute a key immune cell population that actively maintains a tolerogenic milieu in the liver and protects the liver against repeated inflammatory challenges.

## Introduction

Autoimmune hepatitis (AIH) is a chronic liver disease of unknown pathogenesis, characterized by elevated transaminases, abundant polyclonal immunoglobulin G (IgG), and specific autoantibodies. Histological findings include infiltration of the liver with lymphocytes, plasma cells, and macrophages as well as characteristic interface hepatitis [1,2]. Infiltration of T cells and plasmacells as well as a genetic association with certain human leukocyte antigen (HLA) alleles [3] suggest that a dysregulation of adaptive immune responses causes the disease.

The liver provides a unique immunologic environment because several cell types residing within the liver have the capacity to act as antigen-presenting cells (APCs). Dendritic cells [4], Kupffer cells [5], liver sinusoidal endothelial cells (LSEC) [6], hepatic stellate cells [7], but also hepatocytes present antigens and activate CD8 T cells [8]. However, it is controversial whether intrahepatic CD8 T-cell activation results in full effector function [9,10], tolerance by deletion [11], or induction of memory T cells [12].

Previous research demonstrated that priming of naïve CD4 T cells in the liver leads to defective T-cells [13,14] but more recently generation of effector CD4 T cells was also described in mice, in which antigen is expressed in hepatocytes after transfection with a recombinant adeno-associated viral vector [15]. CD4 T cells activated in the liver may alternatively acquire a regulatory phenotype [16], and Foxp3 is induced on CD4 T cells co-cultured with hepatocytes [17], LSEC [18], or hepatic dendritic cells [19].

Treg express the transcription factor Foxp3, mostly originate from the thymus, and support immunological tolerance [20]. Other Treg subtypes are induced in the periphery [21]. Treg inhibit the function of effector lymphocytes or APCs by releasing Interleukin-10 (IL-10) and transforming growth factor beta (TGF-β), by lysing target cells with granzymes, or by consuming cytokines indispensable for effector cells. Treg also form stable interactions with DCs and thereby inhibit activation of effector cells [22].

The role of Treg in autoimmune liver disease is still under debate. Treg in the blood of patients suffering from AIH are numerically reduced and functionally impaired, e.g. fail to suppress secretion of pro-inflammatory IL-17 by effector CD4 T cells [23–26]. Others observed that blood-derived Treg from AIH patients effectively suppress immune responses [27]. An increase of Treg numbers in liver tissue was shown to depend on disease activity [28], suggesting that Treg play an essential role in maintaining liver tolerance.

Studies from animal models support the hypothesis that Treg suppress liver inflammation. Neonatal thymectomy in mice deficient in programmed cell death-1 (PD-1) leads to inflammation of the liver, among other organs, and is explained by the loss of Treg [29]. Transfer of *ex vivo* generated Treg restores tolerance and reduces liver inflammation in a model of type 2 AIH [30]. Treg are required to limit liver damage in mice transduced with a hepatitis B virus genome by adenoviral transfer, but also prolong clearance of transduced hepatocytes [31]. Intramuscular vaccination with hepatitis B antigen (HBsAg) leads to accumulation of antigen-specific CD8 T cells in the liver followed by infiltration of CD4^+^Foxp3^+^ T cells [32]. Likewise, accumulation of Treg is observed in livers of mice suffering from chronic hepatitis [33]. Finally, Treg generated by liver-derived antigen suppress experimental autoimmune encephalomyelitis, suggesting that systemic tolerance is induced by hepatic Treg [18,34].

We analyzed the role of Treg in liver inflammation in a transgenic mouse model of T-cell mediated hepatitis.

## Materials and Methods

### Animals and cells

TF-OVA mice were described before [9]. TF-OVA mice express ovalbumin under the transferrin promoter in hepatocytes. Transfer of naive antigen-specific OT-I T cells into TF-OVA mice leads to their activation in the liver and to transient hepatitis. DEREG (DEpletion of REGulatory T cells) mice [35] (kindly provided by T. Sparwasser) were bred to TF-OVA mice. Treg in TF-OVAxDEREG mice were depleted by i.p. application of 1μg diphtheria toxin (DT) (Sigma-Aldrich) at days -1, 1 and 3 after T-cell transfer and additionally at day 20, 22 and 24 in some experiments.

Naïve CD8 T cells were isolated from lymph nodes and spleen of OT-I mice [36], using isolation kits from Miltenyi Biotec (Bergisch-Gladbach, Germany). Purity of preparations was above 95%. To induce hepatitis, 4x10^6^ CD8 OT-I T cells were injected i.v. into TF-OVA or TF-OVAxDEREG mice.

To isolate intrahepatic lymphocytes, livers were perfused with PBS/0.5% BSA, fragmented, and passed through a 70μm nylon mesh. After brief centrifugation at 300rpm, cells were separated on a discontinuous 40/70% Percoll gradient at 2000 rpm, 20min (Biochrom, Berlin, Germany). Splenic cells were isolated by passing spleens through a 70μm nylon mesh, followed by red blood cell lysis.

All animals received humane care according to institutional criteria. All animal procedures were approved by the Landesamt für Gesundheit und Soziales, Berlin (registration G0191/09).

### In vitro T-cell suppression assay

For antigen specific *in vitro* T-cell suppression assays CD8 OT-I T cells were labelled with 1.5 μM carboxy-fluorescein succinimidyl ester (CFSE, Invitrogen). APCs were isolated from spleens of TF-OVA mice by passing through a 70μm nylon mesh and lysis of erythrocytes. Hepatic Treg were isolated at day 5 from livers of TF-OVA mice suffering from hepatitis, as described above. Hepatic lymphocytes were stained with fluorescent antibodies and sorted for CD4^+^CD25^+^ T cells. Peripheral Treg and naïve CD4 T cells were isolated from lymphoid tissues of C57Bl/6J mice, as described above. Subsequently, cells were stained with fluorescent antibodies and sorted for Treg (CD4^+^CD25^+^) and naïve CD4 T cells (CD4^+^CD25^-^). 5x10^4^ CFSE-labeled CD8 OT-I T cells were seeded in complete RPMI in 96-well plates, stimulated with 1x10^5^ APCs at 37°C alone or together with 5x10^4^ hepatic Treg (liver Treg), peripheral Treg (control-Treg), or naïve CD4 T cells for three days. Proliferation of CD8 OT-I responder T cells was determined by flow cytometry of CFSE-dilution.

### Histology, immunohistochemistry, and immunofluorescence

For histology, livers were perfused with PBS and fixed for 24h in 4% paraformaldehyd, followed by embedding in paraffin. 3μm sections were cut and stained with hematoxylin and eosin.

For immunohistochemistry, paraffin sections were deparaffinated and subjected to heat-induced epitope retrieval using sodium citrate buffer solution (pH 6.0). Slides were rinsed in cool tap water and washed in Tris-buffered saline (pH 7.4) prior to incubation with anti-CD3 (Dako #IR50361- 2) followed by detection employing the Dako REAL™ Detection System, Alkaline Phosphatase/RED, Rabbit/Mouse (Dako). For detection of regulatory T cells, sections were subjected to heat-induced epitope retrieval prior to incubation with FoxP3 antibody (FJK-16s, eBioscience, 1:100) followed by incubation with rabbit anti-rat secondary antibody (Dianova, Hamburg, Germany). For detection, EnVision+ System- HRP Labelled Polymer Anti-Rabbit (Dako) was used. HRP was visualized with diaminobenzidine (Dako) as chromogen. Subsequently, sections were subjected to pressure cooking in order to degrade proteins and incubated with anti-CD3 (Dako #IR50361- 2) followed by donkey anti-rabbit antibody (Dianova, Hamburg, Germany) and the streptavidin alkaline phosphatase kit (Dako). Alkaline phosphatase was revealed by Fast Red as chromogen. Nuclei were counterstained with hematoxylin and slides were coverslipped with glycerol gelatine (Merck). Negative controls were performed by omitting the primary antibody.

For immunofluorescence, frozen sections were air-dried, fixed in acetone for 10min, and incubated with anti-CD8 antibody (Ly-2, eBioscience, 1:50) followed by Alexa Fluor 555-conjugated goat anti-rat antibody (1:100, Invitrogen). Nuclei were counterstained with DAPI (Roche, Mannheim, Germany, 1:1500), and slides were mounted in Fluoromount-G (Southern Biotech, Birmingham, USA). Negative controls were performed by omitting primary antibodies.

Images were acquired using a fluorescence microscope (AxioImager-Z1) equipped with a CCD camera (AxioCam) and processed with Axiovision software (Carl Zeiss, Jena, Germany). To quantify cells, ten visual fields of 0.15 mm^2^ of each liver section were counted using ImageJ.

### Flow cytometry

Lymphocytes were stained with anti-CD4, anti-CD25, anti-IFN-γ (BioLegend); anti-FoxP3, anti-Vα2 (eBioscience), and anti-CD8 (BD-Biosciences). For intracellular IFN-γ-staining, cells were fixed and permeabilized using BD-Cytofix/Cytoperm (BD-Biosciences). Intranuclear staining for FoxP3 was performed using the FoxP3 staining set (eBioscience). Cells were analyzed on a BD FacsCalibur or BD FACS Canto II using the CellQuest and DIVA software. For phenotypic characterization of Treg, 4x10^6^ CD8 OT-I T cells were transferred into TF-OVA mice. Mice were sacrificed at day 5, hepatic and splenic Treg from naïve C57Bl/6J mice were isolated as described above, and CD4+CD25+Foxp3+ cells were analyzed using the following antibodies from BioLegend: anti-CD357 (GITR), anti-CD278 (ICOS), anti-CD279 (PD-1), anti-CD103 (integrin-E), anti-CD304 (neuropilin-1), anti-CD62L (L-selectin), anti-CD152 (CTLA-4), anti-Helios (intracellular stain). Control-Treg were isolated as above and stained with the same antibodies.

### T-cell stimulation and *in vitro* cytolysis assay

For stimulation experiments, 4x10^6^ CD8 OT-I T cells were transferred into TF-OVAxDEREG mice. Mice were sacrificed at day 5, non-parenchymal cells were isolated from liver and spleen, cultured in complete RPMI, and activated in 1μg/ml SIINFEKL. After 1h, 2μg/ml brefeldin-A (Sigma) was added, and cells were analyzed for intracellular IFN-γ after an additional 3h.

For *in vitro* cytolysis assays, 4x10^6^ CD8 OT-I Thy1.1 T-cells were transferred into TF-OVAxDEREG mice. Five days later, Thy1.1^+^ T cells were enriched by PE-Beads (Miltenyi Biotec) and PE-labelled Thy.1.1^+^ T cells were sorted from liver and spleen. Splenocytes from C57Bl/6J mice were labeled with 7.5μM (high) or 0.75μM (low) CFSE and pulsed with 1μg/ml SIINFEKL or left untreated for 1 hour, respectively. 2.5x10^5^ Thy1.1^+^ T cells from spleen and liver were incubated with 5x10^5^ CFSE^high^ SIINFEKL-pulsed and 5x10^5^ CFSE^low^ unpulsed splenocytes. As control labeled splenocytes were incubated alone. After 4 hours, cells were analyzed for CFSE-staining and specific lysis was calculated as follows:

$$100\text{x}\left[1-\left(\%{\text{CFSE}}^{\text{low}}\left(\text{control}\right)/\%{\text{CFSE}}^{\text{high}}\left(\text{control}\right)\right)/\left(\%{\text{CFSE}}^{\text{low}}\left(\text{OT-IThy}1.1\right)/\%{\text{CFSE}}^{\text{high}}\left(\text{OT-IThy}1.1\right)\right)\right].$$

### Alanine aminotransferase (ALT) and bilirubin measurements

Blood was collected into separation tubes and sera were stored at -20°C before automatical analysis on a Modular analyzer (Roche, Germany).

### Statistical analysis

Statistical analyses were performed with GraphPadPrism software (GraphPad Software, San Diego, CA).

## Results

### Endogenous regulatory T cells accumulate in the liver of TF-OVA mice during T-cell mediated hepatitis

We investigated whether the number of CD4 T cells present in liver and spleen changes during CD8 T cell-mediated hepatitis.

**Fig 1:**
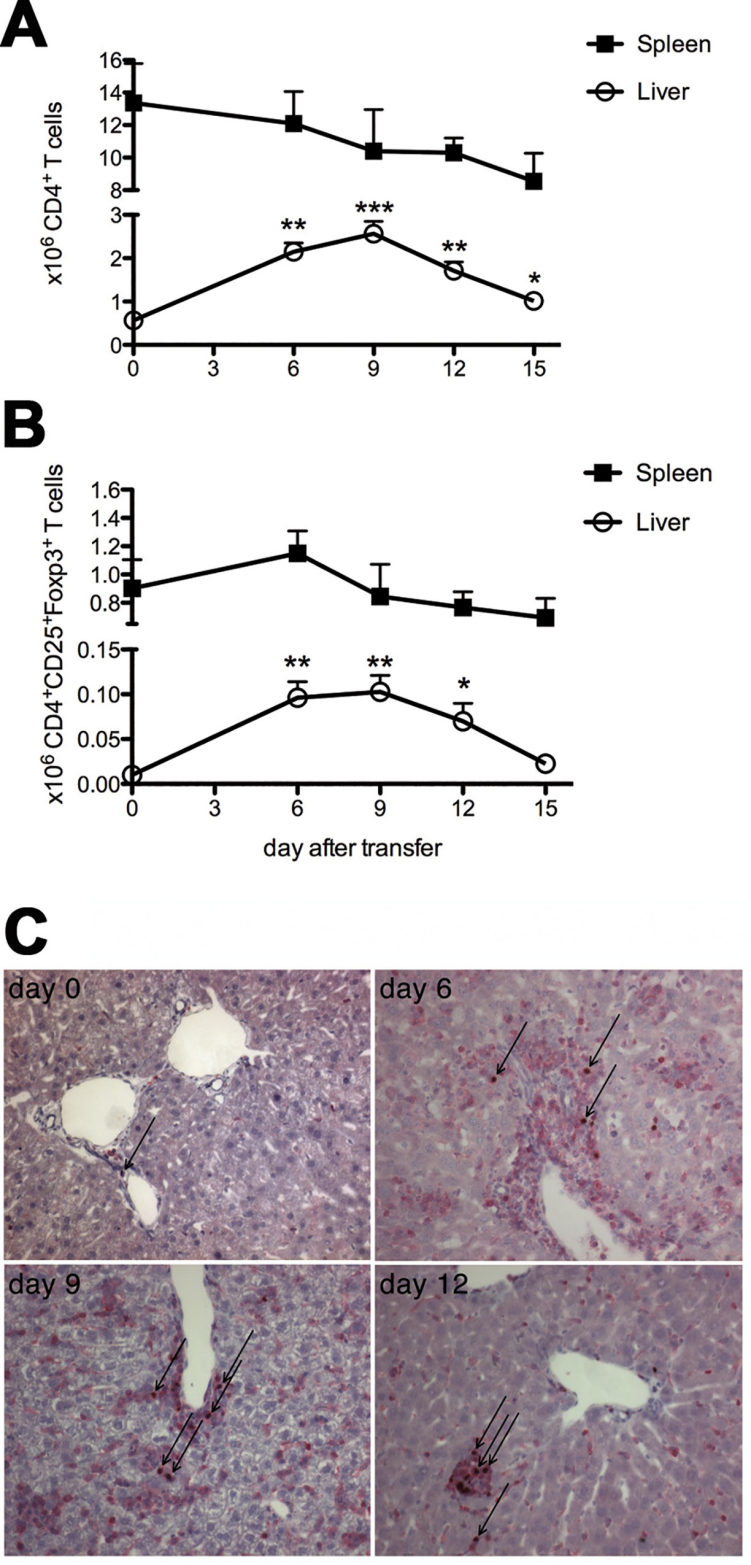
Regulatory T cells accumulate in the liver of mice in the course of hepatitis. 4x10^6^ CD8 OT-I T cells were transferred intravenously into TF-OVA mice. Non-parenchymal cells were isolated from liver and spleen at the indicated days and analyzed for the presence of CD4^+^, CD25^+^, and Foxp3^+^ T cells by flow cytometry. Absolute numbers of (A) CD4^+^ T cells and (B) Foxp3^+^CD25^+^CD4^+^ T cells are depicted (mean±SEM from n = 4–25 mice per time point; ***p<0.001, **p<0.01, *p<0.05 by Mann-Whitney test). (C) Immunohistochemistry for CD3 (red membrane staining) and Foxp3 (brown nuclear staining) of livers from mice at the indicated days after transfer of CD8 OT-I T cells (magnification 200x), arrows indicate exemplary CD3^+^Foxp3^+^ cells). Representative images from 3–4 mice per time point are depicted.

CD4 T-cell numbers increased in livers of TF-OVA mice, reaching their peak nine days after transfer of CD8 OT-I T cells and well after the peak of liver damage (Fig 1A), as determined by ALT levels [9]. We further defined the number of regulatory T cells found within the CD4 T-cell compartment. The number of CD4 T cells expressing CD25 and Foxp3 increased by roughly 10-fold from day zero to day 6/9, and returned to baseline values at day 15 (Fig 1B). In contrast, numbers of CD4^+^ or CD4^+^CD25^+^Foxp3^+^ T cells in the spleen did not significantly change during the course of hepatitis (Fig 1A and 1B, S1 Dataset). Foxp3^+^ cells constituted a sizable percentage of the CD3^+^ T cells infiltrating the portal tracts (Fig 1C).

Thus, regulatory T cells accumulate in the inflamed liver during T-cell mediated hepatitis, suggesting that they play an essential role in limiting autoimmune hepatitis.

### Endogenous hepatic regulatory T cells suppress proliferating CD8 T cells *in vitro*

To analyze whether endogenous Treg, which accumulate in inflamed livers of TF-OVA mice after transfer of CD8 T cells, are functionally active, we performed an *in vitro* T-cell suppression assay. CFSE-labeled CD8 OT-I T cells alone or together with naïve CD4 T cells or CD4 Treg isolated from the livers of TF-OVA mice suffering from hepatitis were stimulated by incubation with spleen cells from TF-OVA mice, which have accumulated the endogenous antigen *in vivo* and are capable of stimulating OT-I T cells [13]. As a control, conventional regulatory T cells isolated from lymph nodes and spleens of wild-type mice were co-incubated with CD8 OT-I T cells.

**Fig 2:**
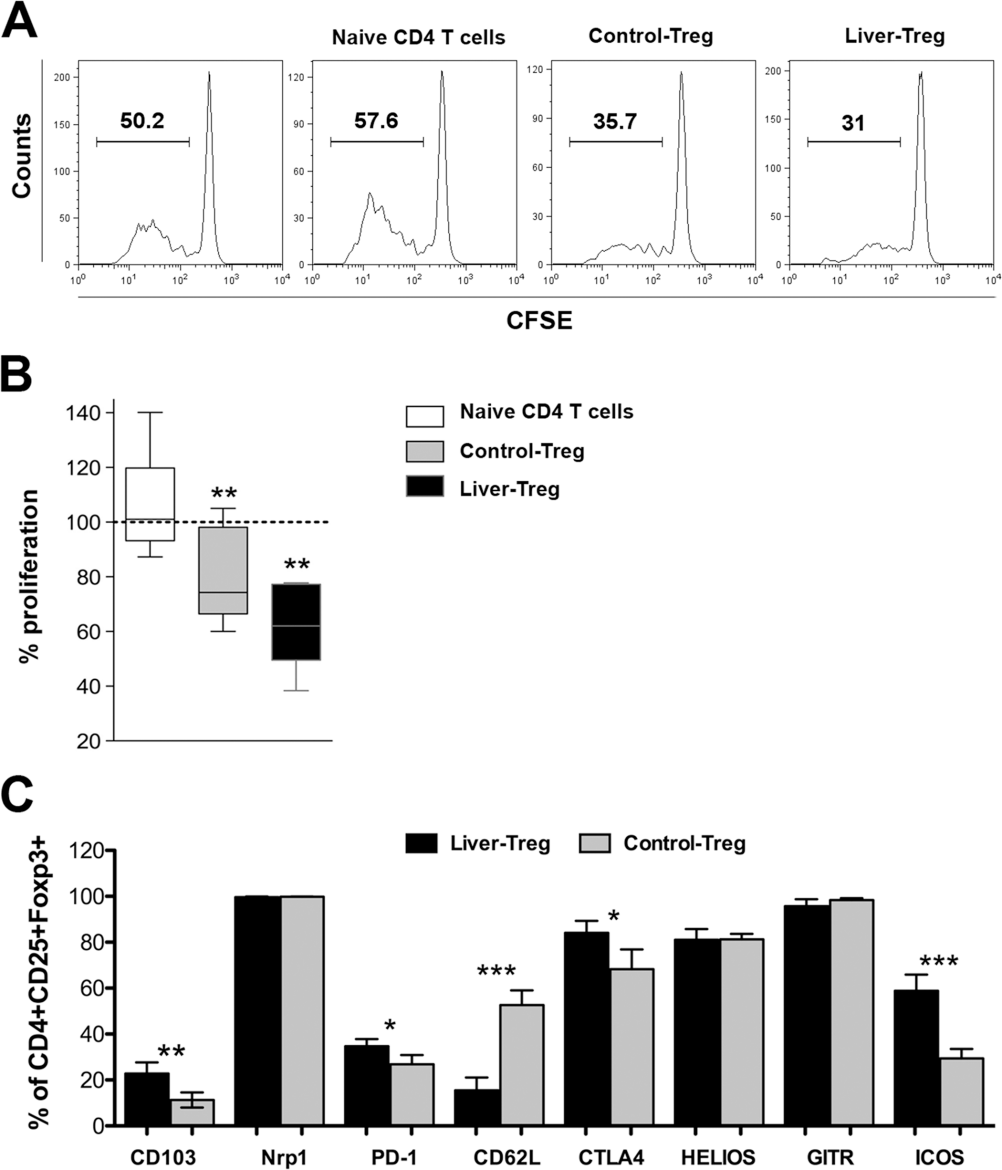
Hepatic regulatory T cells display effector-Treg phenotype and suppress the proliferation of CD8 OT-I T cells *in vitro*. (A) CFSE-labeled CD8 OT-I T cells were stimulated by APCs isolated from spleens of TF-OVA mice for three days alone or in co-culture with CD4^+^CD25^-^ T cells (naïve CD4 T cells), CD4^+^CD25^+^ Treg isolated from lymph nodes and spleens of wild type-mice (control-Treg) or CD4^+^CD25^+^ Treg isolated from livers of TF-OVA mice suffering from hepatitis (liver-Treg). Depicted are representative histograms showing proliferating CFSE-labeled CD8 OT-I T cells at day three, cultured with different CD4 T-cell types (gated on CD8^+^CFSE^+^). (B) The percentage of proliferating CD8 T cell cultured with control-Treg, liver-Treg, or naïve CD4 T cells was calculated by CFSE-intensity relative to the proliferation of CD8 T cells (100% = dotted line) cultured alone. Box plots depict the percentage of CD8 T-cell proliferation. N = 5–9. p-values were calculated using one-sample t-test against a hypothetical value of 100%. **p<0.01. (C) Liver-Treg and control-Treg were generated and isolated as above. CD4^+^CD25^+^Foxp3^+^ cells were analyzed for expression of various markers by flow cytometry. Depicted are mean ± SD from n = 4 mice per group, ***p<0.001, **p<0.01, *p<0.05 by unpaired t-test.

Proliferation was determined by the degree of CFSE-dilution of CD8 OT-I T cells after three days (Fig 2A). Co-culture of CD8 OT-I T cells with naïve CD4 T cells led to slightly higher proliferation of CD8 T cells compared to CD8 T cells alone (Fig 2A and 2B, S2 Dataset), suggesting that CD4 T cells may promote CD8 OT-I T-cell proliferation in this setting. In contrast, addition of Treg isolated from inflamed livers (liver-Treg) or of Treg isolated from lymph nodes of wild-type mice (control-Treg) significantly reduced the proliferation of CD8^+^ responder T cells (Fig 2A and 2B).

We compared the phenotype of liver-Treg isolated from inflamed livers and control-Treg isolated from spleen of naïve C57Bl/6J mice. Both types of Treg expressed neuropilin-1 and Helios, suggesting that both belong to the class of natural Treg derived from the thymus rather than to the class of peripherally induced Treg [37,38]. Liver-Treg displayed stronger expression of ICOS and CD103 (integrin αE) while expression of CD62L was reduced, a phenotype termed effector-Treg, which is found on Treg that populate peripheral tissues [39]. Expression levels of CTLA-4 and PD-1 were also higher in liver-Treg than in control-Treg (Fig 2C, S2 Dataset).

In summary, endogenous regulatory T cells derived from inflamed livers are capable of suppressing CD8 T-cell proliferation *in vitro* to a similar extent as peripheral Treg from wild-type mice. Liver-Treg display the phenotype of effector Treg derived from the thymus.

### Regulatory T cells suppress CD8 T cell-mediated hepatitis *in vivo*

**Fig 3:**
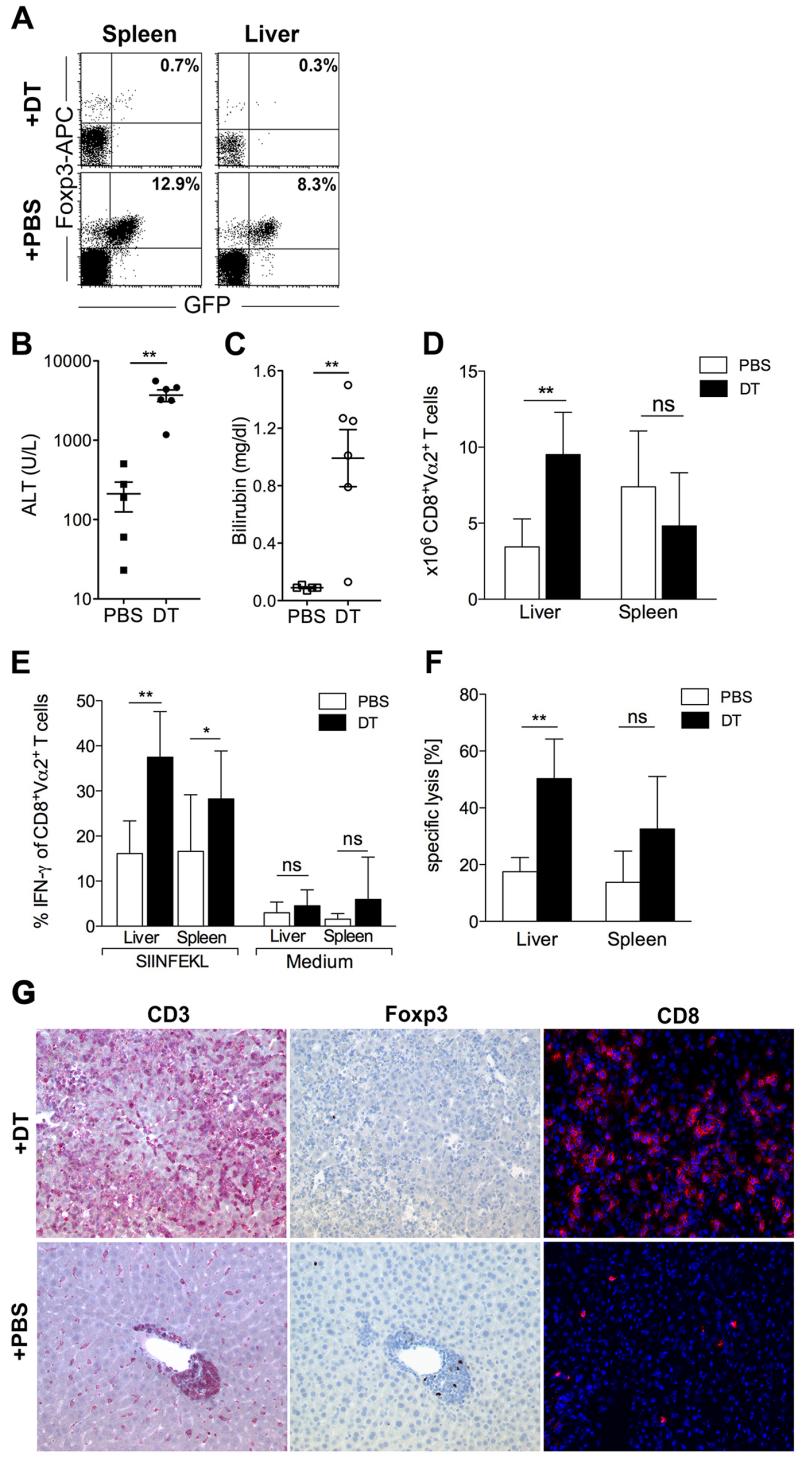
Regulatory T cells suppress CD8 T cell-mediated hepatitis *in vivo*. 4x10^6^ OT-I CD8 T cells were transferred intravenously into TF-OVAxDEREG mice and treated with DT (diphtheria toxin) or PBS as described in methods. (A) Spleens and livers were analyzed for the presence of Foxp3^+^ T cells on day 5. Plots depict data gated on CD4^+^T cells. ALT (B) and bilirubin (C) were measured in serum on days 4 or 5, respectively. Values from individual mice and mean±SEM are depicted. (D) The number of OT-I T cells was estimated by staining for the Vα2 chain, which forms the OT-I TCR, since no clonotypic antibody is available. The number of CD8^+^Vα2^+^ T cells in liver and spleen was determined by flow cytometry. Depicted are mean±SD from 6–7 mice per group. (E) At day 5 after induction of hepatitis, IFN-γ production of CD8 OT-I T cells isolated from liver and spleen was measured after *in vitro* cultivation in medium or SIINFEKL. Plots depict mean±SD, events are gated on CD8^+^Vα2^+^T cells, data are from 5–7 mice per group. (F) 5 days after the induction of hepatitis, OT-I CD8 Thy1.1^+^ T cells were purified from liver and spleen and specific lysis was analyzed and calculated as described in methods. Plots depict mean±SD from 5 mice per group. (G) Histology was assessed on day 4 using antibodies against CD3, Foxp3 (magnification 100x), and CD8 (magnification 200x). Representative results from 5–6 mice in each group are shown. Statistics in (B-F) were performed using the Mann-Whitney-test; ***p<0.001, **p<0.01, *p<0.05; ns = not significant.

Treg numbers increase upon inflammation in the liver in our model and Treg isolated from inflamed livers suppress CD8 T-cell proliferation *in vitro*. These data suggest that they are essential to suppress T cell-mediated hepatitis *in vivo*, which was tested by selective depletion of Treg in mice suffering from T-cell mediated hepatitis. To this end, TF-OVA mice were crossed to DEREG mice, in which application of diphtheria toxin (DT) effectively depletes Treg (Fig 3A and 3G). Transfer of CD8 OT-I T cells led to more severe hepatitis in TF-OVAxDEREG mice treated with DT than in mice treated with PBS, as determined by serum levels of ALT and bilirubin, respectively (Fig 3B and 3C, S3 Dataset). The number of CD8^+^Vα2^+^T cells infiltrating the livers of Treg-depleted mice suffering from hepatitis was significantly higher than in mice that possessed Treg, whereas numbers of CD8^+^Vα2^+^T cells were similar in the spleens of Treg-depleted and Treg-competent mice (Fig 3D, S3 Dataset). We then investigated whether Treg influence the effector function of transferred CD8 OT-I T cells. Depletion of Treg increased the level of IFN-γ secreted by CD8 OT-I T cells isolated from liver and spleen of mice suffering from hepatitis (Fig 3E, S3 Dataset). Specific lysis of target cells by OT-I T cells isolated from the liver was increased by about twofold in mice depleted of Treg compared to mice that were in possession of Treg (Fig 3F, S3 Dataset). In contrast, depletion of Treg had no significant influence on the cytolytic activity of OT-I T cells isolated from the spleen. Histological assessment confirmed that significantly more CD8 T cells infiltrated the livers of mice suffering from hepatitis in the absence of Treg and also confirmed that depletion of CD3^+^Foxp3^+^ T cells upon DT-application was effective (Fig 3G).

Taken together, Treg reduce the number of cytotoxic CD8 OT-I T cells in the liver of mice suffering from immune-mediated hepatitis and are essential to control T cell-mediated hepatitis *in vivo*.

### Regulatory T cells maintain tolerance in CD8 T cell-mediated hepatitis

The transfer of naïve CD8 OT-I T cells into TF-OVA mice results in transient hepatitis characterized by a peak in ALT levels at days 5 to 7 and a return to normal values by day 14 [9]. The fact that mice completely recover from hepatitis within two weeks could be explained by deletion of liver-activated CD8 T cells by apoptosis, as suggested by Bertolino et al. [40]. An alternative mechanism could involve an active mechanism of tolerance. To analyze whether Treg induce tolerance towards liver antigen, we induced hepatitis in TF-OVAxDEREG mice by transfer of CD8 OT-I T cells. After mice had completely recovered from hepatitis, we transferred CD8 OT-I T cells a second time.

**Fig 4:**
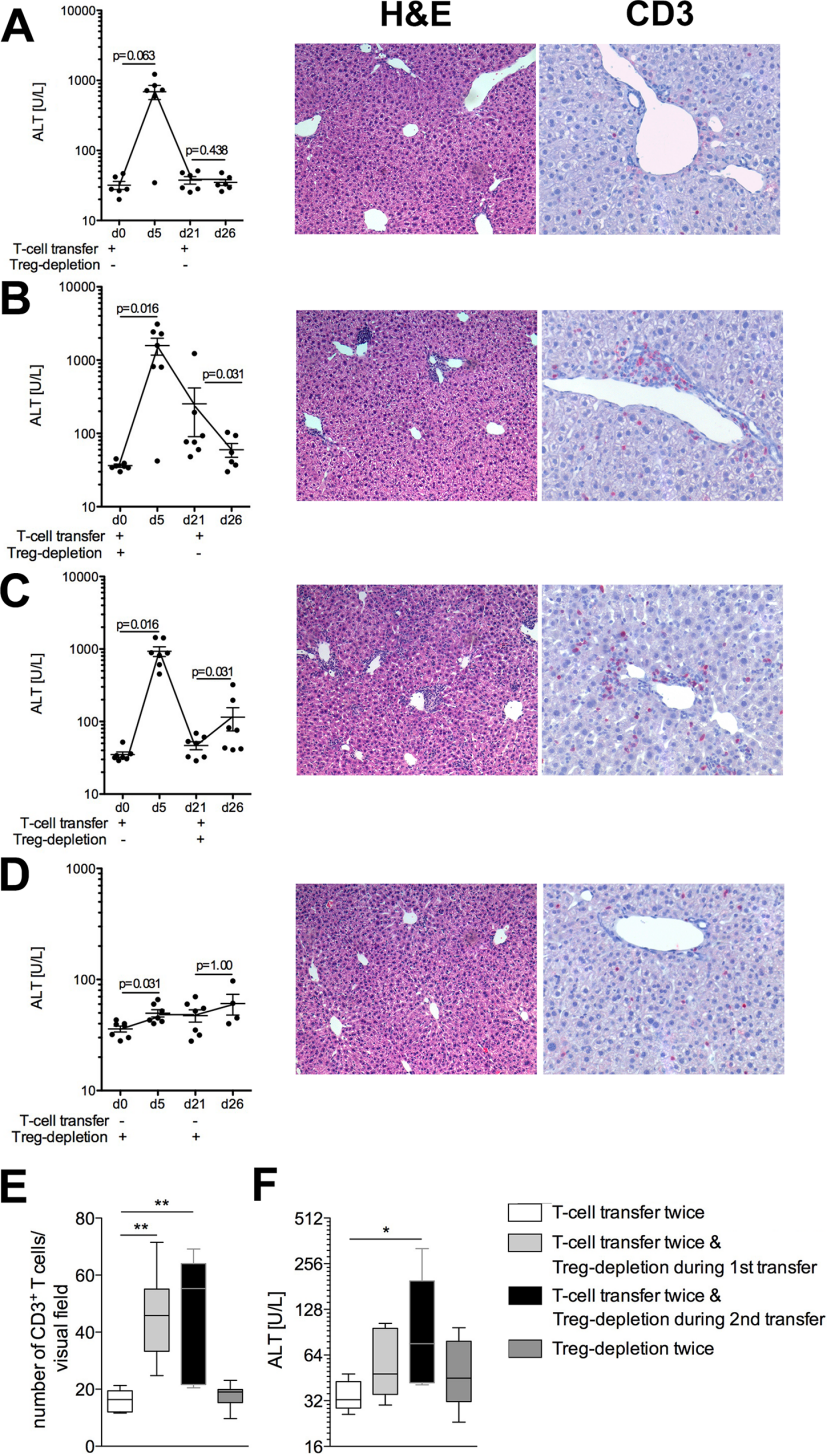
Regulatory T cells maintain tolerance during T-cell mediated hepatitis. 4*10^6^ CD8 OT-I T cells were transferred into TF-OVAxDEREG mice at day 0 and day 21 (A–C). In addition, Treg were depleted by application of DT, either at the time point of the first transfer of CD8 OT-I T cells (B) or at the time point of the second CD8 OT-I T-cell transfer (C). Control mice received DT alone at both time points (D). Depicted are serum ALT levels of mice at the indicated time points. Statistics were performed using the Wilcoxon-signed-rank-test; p-values<0.05 were considered significant. (A-D) Histology of livers was assessed at day 26 using H&E staining (magnification 100x), as well as antibodies against CD3 (magnification 200x; red membrane staining). Depicted are representative pictures from TF-OVAxDEREG mice treated as indicated in A-D. (E) Box plot depicts the number of CD3^+^T cells in liver sections quantified as described in methods of n = 6–7 mice. (F) Box Plot shows the corresponding ALT-levels measured in sera of n = 5–7 mice at day 26. *p<0.05, **p<0.01; Kruskal–Wallis with Dunn’s posttest.

As expected, transfer of CD8 OT-I T cells into TF-OVAxDEREG mice led to liver damage, indicated by increased ALT levels at day five and recovery by day 21 (Fig 4A). The second transfer of CD8 OT-I T cells induced no further liver damage as indicated by unaltered ALT levels at day 26, i.e. day 6 after the second T-cell transfer. Likewise, no infiltration of T cells into the liver was detected (Fig 4A and 4E, S4 Dataset). This result suggests that an active mechanism of tolerance was established in the course of hepatitis. Therefore, we further investigated whether Treg are involved in liver tolerance by depleting Treg either during the first or during the second round of CD8 T-cell transfer.

Treg-depletion during the first T-cell transfer led to more severe hepatitis, as indicated by further increased ALT levels (Fig 4B, S4 Dataset). Although ALT levels declined, some mice did not completely recover from hepatitis by day 21 in the absence of Treg. However, a second injection of CD8 OT-I T cells at day 21 did not increase ALT levels again (Fig 4B and 4F, S4 Dataset). Remnant T-cell infiltrates were detected in liver-sections at day 26 (Fig 4B and 4E, S4 Dataset). Thus, the absence of Treg during the initial T-cell transfer led to more severe hepatitis and slower recovery from liver inflammation compared to mice that were in possession of Treg. Nevertheless, a tolerogenic response was re-established by day 21.

Next, CD8 OT-I T cells were transferred at days 0 and 21, and Treg were depleted at the time of the second T-cell transfer (Fig 4C, S4 Dataset). As expected, the first T-cell transfer led to elevated ALT at day five which had resolved by day 21. Depletion of Treg during the second round of T-cell transfer led to a moderate increase in ALT levels at day 26, i.e. 6 days after the second T-cell transfer (Fig 4C and 4F, S4 Dataset). Although the rise in ALT levels was lower than the one observed after the first round of T-cell transfer and was not observed in all mice, it was statistically significant and was accompanied by a sizable T-cell infiltration in the liver (Fig 4Cand 4E, S4 Dataset). While control mice receiving DT alone in the absence of T cells showed a slight increase of ALT values (Fig 4D, S4 Dataset), no T-cell infiltration was noted at day 26 (Fig 4D and 4E, S4 Dataset).

Together, these results show that an active mechanism of tolerance is involved in the clearance of hepatitis and suggest that Treg may be required to maintain tolerance in the liver.

## Discussion

Patients with AIH exhibit T-cell infiltrates in the liver that include both CD8 and CD4 T cells [41,42]. It is still debated to what extent these T cells contribute to the pathogenesis of AIH. In our model antigen-specific CD8 T cells are primed in the liver by various hepatic cell types, such as hepatocytes and LSEC, but also by professional APCs [9,13]. The quality of CD8 T-cell activation in the liver depends on the type of APC. In TF-OVA mice professional APCs are indispensable for CD8 T cells to gain full effector function. We and others have reported on the failure of CD4 T cells to be activated by liver-derived antigen in the liver, even under inflammatory conditions [13,14]. Induction of hepatitis in TF-OVA mice leads to transient hepatitis lasting approximately 14 days [9]. The mechanisms resulting in the resolution of hepatitis is elusive. The data shown here strengthen the notion that Treg play an essential part in suppressing the activity of antigen-specific CD8 T cells in the liver, thereby ameliorating T cell-mediated hepatitis.

We found that Treg accumulate in the liver corresponding to the expansion of CD8 OT-I T cells and to the degree of liver inflammation [9], confirming data from previous reports [32,33]. The enrichment of Treg in the liver during the course of hepatitis suggests an essential role in regulating liver inflammation. Indeed we show that hepatic Treg suppress CD8 T-cell activation *in vitro* to a similar degree as peripheral Treg isolated from secondary lymphoid organs, indicating that Treg isolated from inflamed liver tissue retain their suppressive potential, at least in our mouse model. While we did not address the mode of action of inhibition in our model, the fact that liver-Treg express neuropilin-1 and Helios suggests that they are thymus-derived and likely employ the same mechanisms as conventional natural Treg. In contrast to control-Treg isolated from spleen liver Treg displayed the effector Treg phenotype typical for Treg found in peripheral tissues [39].

Non-functional Treg were observed in the peripheral blood in patients suffering from AIH [23,26] while functional Treg are observed in our model. This discrepancy could be explained by the fact that peripheral Treg may not be representative of liver-infiltrating Treg or could result from the chronic condition that the patients are suffering from and that is clearly different from the transient hepatitis induced in our model.

As of now, it is unknown whether Treg infiltrating inflamed livers of TF-OVA mice have immigrated to the liver from secondary lymphoid tissues or were induced within the liver itself. Our data argue against an origin in the spleen since Treg-numbers remained stable in the spleen during hepatitis. While *in vitro* experiments indicate that different hepatic cell types such as DCs [19], stellate cells [43], hepatocytes [17], LSECs, and KCs [18] induce functional CD4^+^Foxp3^+^ T cells, evidence for *in vivo* induction of Treg in the liver remains scarce. Lüth et al. reported induction of myelin basic protein (MBP)-specific Treg in mice expressing MBP in hepatocytes [34]. These Treg were capable of inducing systemic tolerance in mice in a model of experimental autoimmune encephalitis. Since Treg were isolated from both liver and spleen it cannot be concluded from this study that Treg were primed within the liver. In our model conversion of naïve transgenic OT-II CD4 T cells into Foxp3^+^ T cells in the liver *in vivo* is an extremely rare event [13], an observation that may be attributable to the fact that OT-II CD4 T cells express a transgenic TCR and may be incapable of differentiating into Treg. At this time, both mechanisms may contribute to immune homeostasis in the liver: maintenance of tolerance through induction of Treg in the liver and recruitment of Treg into the liver driven by chemokines induced during liver inflammation [44]. However, the fact that liver-Treg display an effector-Treg phenotype suggests that they originate from central-Treg residing in lymph nodes and have migrated to the liver after activation and proliferation [39].

We observe increased infiltration of antigen-specific CD8 OT-I T cells in the liver in the absence of Treg. In addition, infiltrating CD8 T cells display improved effector function, i.e. produce more IFN-γ and display increased cytolytic function leading to more severe hepatitis. Similar results were observed in a mouse model of HBV infection, in which depletion of Treg likewise increased liver damage but at the same time improved clearance of HBs-Ag positive hepatocytes [31]. However, this model relies on adenoviral transfer of the antigen to hepatocytes. Therefore the antigen is solely presented on MHC I molecules and activation of innate immune responses is likely, possibly altering the activity of Treg (as reviewed in [45]). In contrast, an endogenous antigen is expressed by hepatocytes and presented as well as cross-presented by professional APCs in our model [9].

Zierden et al. observed accumulation of Treg in a transgenic mouse model of chronic liver inflammation. Although the consequence resulting from depletion of Treg in the chronic phase of the disease was not investigated, their results suggest that Treg may be capable of regulating the immune response in the acute phase of inflammation but much less so in chronic inflammation [33].

Our data add to the understanding of the role of Treg during liver inflammation by confirming that depletion of Treg increases liver damage in acute hepatitis in a model of T- cell immunity against a self antigen and extends it by demonstrating that Treg induced during the initial inflammatory event persist and protect from further immune attacks. A second transfer of CD8 OT-I T cells after remission of hepatitis failed to induce an inflammatory response in the liver, suggesting that the first round of T-cell activation in the liver had skewed the environment in the liver towards tolerance. Depletion of Treg during the first or the second CD8 T-cell attack led to increased T-cell infiltration and release of ALT, suggesting that Treg maintain the tolerant milieu in the liver to some degree.

Our data suggest that depletion of liver-activated T cells by default is not the only factor contributing to tolerance and rather suggest that Treg are actively involved in the induction of tolerance in the liver to some degree. Independently of the timing of Treg-depletion, the second CD8 T-cell response did not reach the magnitude of the first response, suggesting that besides Treg other, yet unidentified, mechanisms that induce tolerance play a significant role in the liver.

Cytokines with immunmodulatory function such as IL-10 and TGF-β are involved in induction of tolerance in Con-A-mediated hepatitis [46,47] and may as well play a role in our model. A specific regulatory T-cell population, which shows attributes of memory T cells, is induced in mice upon inflammation in the skin [48]. These “memory Treg” reside within tissues and are more effective in suppressing a secondary immune response, a possible mechanism explaining induction of tolerance in the liver in our model.

In summary, our data confirm that Treg accumulate in the liver in the course of immune-mediated hepatitis in our model, limit the severity of hepatitis and actively induce tolerance, which protects against a second attack by T cells primed by the same antigen.

## Supporting Information

S1 Dataset Raw data supporting Fig 1. (XLSX)

S2 Dataset Raw data supporting Fig 2. (XLSX)

S3 Dataset Raw data supporting Fig 3. (XLSX)

S4 Dataset Raw data supporting Fig 4. (XLSX)
